# Peri-implantitis derived extracellular vesicle as vectors of neuroinflammation

**DOI:** 10.3389/fncel.2026.1773682

**Published:** 2026-03-03

**Authors:** Gestter Willian Lattari Tessarin, Rodrigo Martins dos Santos, Luan Felipe Toro

**Affiliations:** 1 School of Dentistry, University Center in the North of São Paulo (UNORTE), São José Do Rio Preto, São Paulo, Brazil; 2 Department of Basic Sciences, School of Dentistry, Sao Paulo State University (UNESP), Araçatuba, São Paulo, Brazil; 3 Integrated Colleges of Três Lagoas (AEMS), Três Lagoas, Mato Grosso do Sul, Brazil; 4 Department of Basic Subjects, Marília Medical School (FAMEMA), Marília, São Paulo, Brazil

**Keywords:** central nervous system, extracellular vesicles, microorganisms, neuroinflammation, peri-implantitis

## Introduction

Peri-implantitis (PI) results from biofilm accumulation on implant-supported crowns and/or implants coils, triggering an immunoinflammatory response that compromises peri-implant tissues (Scarano et al., 2023). PI and periodontitis (PD) share marked similarities in their microbiological profiles, pathogenesis, disease progression, and immune-inflammatory responses (Tessarin et al., 2024). Key pathogens, such as *Porphyromonas gingivalis, Treponema denticola*, and *Tannerella forsythia*, are frequently identified in both conditions (de Waal et al., 2017; Tessarin et al., 2024) and can release extracellular vesicles (EVs; Schuh et al., 2019). Recent evidence has highlighted that both PI and PD are associated with the development of systemic diseases, including cancer, cardiovascular and brain disorders, and type 2 diabetes (Bui et al., 2019; Sansores-España et al., 2021; Cafferata et al., 2024; Tessarin et al., 2024). In PD, EVs released by microorganisms have been shown to reach the brain and contribute to neuroinflammation (Lee et al., 2023; Butler et al., 2024; Zhang et al., 2025). Considering the shared etiological factors and host responses between PD and PI, this analysis examined whether EVs released during PI may act as triggers or amplifiers of neuroinflammation.

## Similarity between periodontal disease (PD) and peri-implantitis (PI): a brief report

PI and PD affect the supporting tissues surrounding dental implants and teeth, respectively. Both pathologies are induced and maintained by dysbiosis between microorganisms and host immunoinflammatory cells (Parga et al., 2024). Studies have reported that several microorganisms observed in PD are also found in PI, such as *Porphyromonas gingivalis, Treponema denticola, Tannerella forsythia*, and *Fusobacterium nucleatum*, among others (Maruyama et al., 2014; Ata-Ali et al., 2015; Rajasekar and Varghese, 2023). Immunological and inflammatory studies have revealed that PD is characterized primarily by increased infiltration of neutrophils and lymphocytes, followed by the recruitment of macrophages to the affected sites. Similarly, PI exhibits elevated concentrations of B cells, neutrophils, and macrophages (Carcuac and Berglundh, 2014; Kinane et al., 2017). Recently, Malmqvist et al. (2024), using soft tissues and crevicular fluid from human subjects, observed that immune cell composition did not differ between PI and PD. In addition, interleukin-1β (IL-1β), interleukin-6 (IL-6), and tumor necrosis factor-alpha (TNF-α) are key pro-inflammatory cytokines implicated in both PD and PI, driving tissue destruction and bone loss (Guarnieri et al., 2024; Tessarin et al., 2024).

## Extracellular vesicles and systemic inflammation

EVs, which are released by cells and microorganisms (Schuh et al., 2019), are composed of bioactive molecules, including proteins, lipids, RNA, DNA, and others (Cabrera-Pastor, 2024). Studies have shown that PD can also induce/potentiate neuroinflammation through microorganisms and/or their products, such as EVs (Zhang et al., 2024, 2025). Recent evidence indicates that EVs are key players in the pathogenesis of inflammatory diseases, as they carry immunogenic molecules recognized by host receptors, thereby triggering pathological inflammation (Xie et al., 2023; Catalan et al., 2024). Intravenous infusion of EVs has been shown to induce strong proinflammatory activity, upregulating cytokine-, chemokine-, and reactive gene expression (Kodali et al., 2024). Moreover, exosomes derived from lipopolysaccharide (LPS) elevated multiple proinflammatory cytokines in mice, suggesting that they can transport inflammatory signals from the periphery to the central nervous system (CNS), thereby inducing neuroinflammation (Kodali et al., 2024).

The EVs-mediated gut–brain axis has been discussed (Uceda et al., 2025; Benameur et al., 2025). EVs from immune cells and the intestinal epithelium under dysbiotic conditions have been shown to cross the blood–brain barrier (BBB) and elicit neuroinflammatory responses within the CNS (Uceda et al., 2025). Exosomes enriched with LPS appear to activate toll-like receptors (TLRs) on microglia, thereby promoting a persistent proinflammatory state (Uceda et al., 2025). In models overexpressing α-synuclein, substantial aggregation of this protein has been observed in the brains of conventional mice compared with germ-free counterparts. Moreover, germ-free mice receiving oral administration of specific bacterial metabolites exhibited a significant increase in neuroinflammation, indicating that the gut microbiota and its secreted components, such as EVs, may play a critical role in α-synuclein pathology and microglial activation (Sampson et al., 2016).

Teng et al. (2022) demonstrated that isoamylamine contributes to neurodegeneration by inducing microglial cell death, possibly reaching the brain through increased intestinal permeability caused by dysbiosis. Collectively, these findings highlight how microbial metabolites, including EVs, can influence neuroinflammation.

## Discussion

### Peri-implantitis and brain inflammation

PD can induce and/or potentiate neurological diseases (Bui et al., 2019; Gil-Montoya et al., 2025; Huang et al., 2025). However, the number of studies reporting an association between PI and neurological disorders remains very limited. Tessarin et al. (2024) reported that microorganisms originating from PI and their products can enter the bloodstream, alter the BBB permeability, and stimulate macrophages and endothelial cells to release proinflammatory mediators that activate astrocytes and microglial cells, thereby promoting neuroinflammation. Trigeminal nerve fibers express receptors, such as TLRs, that recognize LPS and other microbial components, activating NF-κB signaling and inducing the release of IL-1β, IL-6, and TNF-α from trigeminal neurons (Tessarin et al., 2024). In addition, certain microorganisms can inhibit phagolysosome formation within neurons, allowing intracellular survival and sustained cytokine release, which in turn activates microglia and astrocytes (Tessarin et al., 2024). Cafferata et al. (2024), using a PI model, demonstrated high levels of IL-6 and TNF-α, along with increased expression of the neuroinflammatory markers GFAP and IBA-1 in the hippocampus, indicating microgliosis and astrocytosis commonly associated with neuroinflammation.

### Extracellular vesicles from microorganisms in PI and brain inflammation

**Figure 1:**
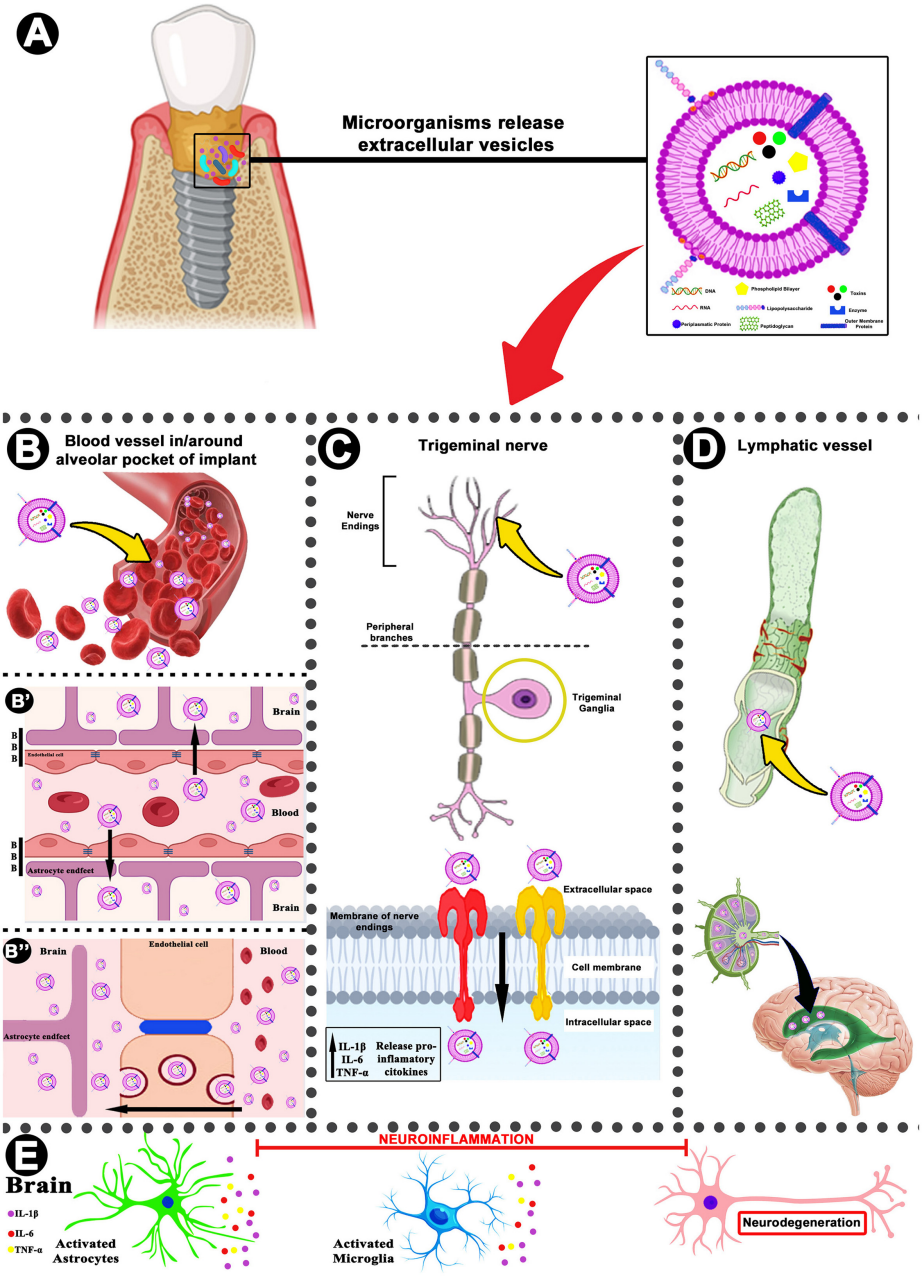
Schematic representation of the proposed pathways by which extracellular vesicles (EVs) released in inflamed peri-implant tissues may induce neuroinflammation. In **(A)**, presence of microorganisms within peri-implant tissues are capable of releasing EVs rich nucleic acids, virulence-associated proteins, toxins, enzymes, and other components. In **(B)**, EVs may enter local blood vessels. In **(B′)**, the EVs can reach from local blood vessels to cerebral vasculature, disorganize the BBB permeability and enter in the parenchyma/stroma of the CNS promoting microglial activation, reactive astrogliosis, and subsequent neuronal dysfunction. In **(B″)**, the transcytosis (internalized EVs via endocytosis at one plasma membrane surface, transported across the cell in membrane-bound vesicles, and released by exocytosis in epithelial cells) also can induced microglial activation, reactive astrogliosis, and subsequent neuronal dysfunction. In **(C)**, trigeminal nerve fibers express multiple receptor types, including TLRs, which can recognize EVs. Activation of neurons within the trigeminal ganglion promotes the release of proinflammatory cytokines such as IL-1β, IL-6, and TNF-α. Sustained cytokine production may extend to the trigeminal ganglia or other brain regions, triggering microglial and astrocytic activation and initiating a neuroinflammatory cascade. In **(D)**, EVs may also migrate from the oral cavity to the brain through lymphatic pathways, particularly at the level of the fourth ventricle, contributing to neuroinflammation. In **(E)**, collectively, these mechanisms may culminate in neuroinflammation, neuronal dysfunction, and neuronal cell death. BBB, blood–brain barrier; TLRs, toll-like receptors.

Bacterial extracellular vesicles (BEVs) are membrane-bound structures composed of a phospholipid bilayer, with diameters ranging from 20 to 400 nm (Devati et al., 2025). They mainly comprise outer membrane vesicles (OMVs) derived from Gram-negative bacteria and membrane vesicles (MVs) released by Gram-positive bacteria (Devati et al., 2025). These vesicles are typically enriched with a variety of biomolecules, including nucleic acids, virulence-associated proteins, toxins, and other components, which underpin their essential roles in biomolecule transport, intercellular communication, and microbial pathogenesis (Devati et al., 2025; Di Naro et al., 2025; Figure 1A). Studies have explored the interconnection between BEVs and inflammatory diseases (Peregrino et al., 2024; Zubair et al., 2024; Lei et al., 2025). Extensive analyses have examined the relationships among gut dysbiosis, PD, apical periodontitis, and neuroinflammation, suggesting the existence of a “gut–mouth–brain axis” (Grenham et al., 2011; Shen et al., 2012; Bui et al., 2019; Xu et al., 2023; Li et al., 2024; da Conceição Francisquini et al., 2025). However, evidence linking PI to neurological diseases remains scarce. Epidemiological data indicate that PD is associated with systemic diseases (Bui et al., 2019), and recent findings suggest that pathogenic nanoparticles can disseminate from periodontal sites to distant tissues, thereby contributing to the development and/or potentiation of systemic illnesses (Kodali et al., 2024).

PI and PD share similarities in their inflammatory responses, characterized by neutrophil recruitment and the release of proinflammatory cytokines, such as IL-1β and TNF-α, as well as matrix metalloproteinases (MMPs) that mediate tissue degradation (Alves et al., 2022; Di Spirito et al., 2024). PI is characterized by a complex microbial ecosystem predominantly composed of *Porphyromonas gingivalis, Tannerella forsythia*, and *Treponema denticola* (Ma and Cao, 2021). Subsequent studies have expanded the spectrum of pathogenic microorganisms to include Gram-negative species, such as *Aggregatibacter actinomycetemcomitans* and *Fusobacterium nucleatum* (Di Spirito et al., 2024), as well as *Streptococcus* spp., *Filifactor alocis*, and others (Diaz et al., 2016; Arenas Rodrigues et al., 2018). These peri-implant pathogens and periodontopathogens harbor a wide array of virulence factors, among which bacterial membrane-derived vesicles have attracted increasing attention (Ma and Cao, 2021). Thus, membrane-derived vesicles released by microorganisms involved in PD and gut microbiota dysbiosis can induce neuroinflammation and contribute to neurodegenerative diseases (Ma and Cao, 2021; Li et al., 2024; Kong et al., 2025; Papadakis et al., 2025). Based on this evidence, it is possible to infer that similar extracellular vesicles, likely released by microorganisms involved in PI, may also contribute to disturbances of the CNS, including inflammation and other pathological conditions (Figures 1A–E).

*Porphyromonas gingivalis* produces virulence factors known as gingipains (Tubero Euzebio Alves et al., 2024), which can be secreted into the extracellular milieu or associated with EVs (Dominy et al., 2019). In post-mortem brain tissue from patients with Alzheimer's disease, gingipains have been detected in the hippocampus and cerebral cortex (Dominy et al., 2019). In murine models, EVs derived from *Aggregatibacter actinomycetemcomitans* and injected intracardially were subsequently detected in the brain, promoting increased TNF-α expression and suggesting OMVs-induced neuroinflammation (Han et al., 2019), contributing to neuronal cell death (Rompikuntal et al., 2012; Aguayo et al., 2018). Since these two microorganisms can also be found in PI, it is possible that similar mechanisms may contribute to the onset and/or potentiation of neuroinflammatory conditions.

Another important point that warrants discussion is the ability of EVs to cross the BBB and induce/potentiate neuroinflammation (González-Sanmiguel et al., 2020; Shawkatova et al., 2025). The BBB is composed of microvascular endothelial cells that line the cerebral capillaries supplying the brain and spinal cord in most mammals and other organisms with a well-developed CNS (Kadry et al., 2020). The BBB plays a pivotal role in regulating the influx and efflux of biological substances essential for metabolic activity and neuronal function (Cunha et al., 2024). Alterations in BBB permeability can occur when pathogens associated with PD and their toxins are recognized by endothelial receptors, such as Toll-like receptors 2 (TLR2) and 4 (TLR4), thereby activating inflammatory cascades (Lei et al., 2023; Ochoa et al., 2025). This recognition stimulates the release of cytokine networks that induce a complex proinflammatory and prothrombotic phenotype in endothelial cells (Li et al., 2022). For example, TNF-α and IL-1 promote the upregulation of chemokines and adhesion molecules, including intercellular adhesion molecule-1 (ICAM-1) and vascular cell adhesion molecule-1 (VCAM-1; Li et al., 2022). In addition, *Porphyromonas gingivalis* can release gingipains capable of degrading extracellular matrix components, thereby penetrating deeper layers of arterial or oral endothelial tissues and establishing colonization (O'Brien-Simpson et al., 2009; Wilensky et al., 2013). Furthermore, ICAM-1 can interact with fibrinogen and reduce the expression of actin-associated tight junction proteins, including occludin and zonula occludens-1, resulting in increased endothelial permeability (Leite et al., 2020; Wang et al., 2023). Endothelial activation also involves nuclear factor kappa B (NF-κB) signaling, leading to the secretion of proinflammatory cytokines that promote macrophage migration and chemotaxis (Leite et al., 2020). In addition, the study carried out by Xie et al. (2023) demonstrated that *Helicobacter pylori* EVs translocate from the stomach to the brain through transcellular pathways without disrupting the gastrointestinal epithelium or the BBB, a phenomenon also observed by Qiu et al. (2025) for *Porphyromonas gingivalis* and referred to as transcytosis (Xie et al., 2023). As discussed above, the microenvironments observed in PD and PI share certain similarities in terms of microbial composition and other characteristics (Di Spirito et al., 2024; Malmqvist et al., 2024; Tessarin et al., 2024). However, to our knowledge, no experimental studies have yet explored potential BBB disruption and/or increase of permeability under PI conditions. Nevertheless, given that these two diseases share common factors, it is plausible to hypothesize that BBB alterations described in PD may also be extrapolated to PI (Figures 1B, B′, B″).

Associated alterations in intracellular calcium disrupt endothelial tight junctions and drive the secretion of MMPs, which further degrade the basal lamina and enhance vascular permeability (Konradt and Hunter, 2018). These processes facilitate the translocation of microorganisms and EVs into the bloodstream, enabling their dissemination to distant tissues. Notably, similar endothelial alterations have been reported in vasculature outside the oral cavity, including the BBB (Konradt and Hunter, 2018). In this context, Lei et al. (2023), using both *in vivo* and *in vitro* analyses, demonstrated that bacteremia induced by *Porphyromonas gingivalis* increased BBB permeability by upregulating caveolin-1 (Cav-1) expression and inhibiting the major facilitator superfamily domain-containing 2a (Mfsd2a). The Cav-1/Mfsd2a complex plays a critical role in regulating BBB permeability. Furthermore, EVs derived from the microbiome carry a diverse array of bioactive compounds capable of influencing CNS function by modulating multiple signal transduction pathways, ultimately contributing to neuroinflammation (Wang et al., 2023).

Finally, the possibility that EVs activate TLRs and traffic through lymphatic vessels cannot be ruled out. Fibers of the trigeminal nerve express receptors such as TLR2 and TLR4, which can be activated by BEVs (Liu et al., 2024). This activation triggers intracellular signaling cascades and enhances NF-κB transcriptional activity (among other pathways), leading to increased release of pro-inflammatory cytokines, including IL-1β, IL-6, and TNF-α from trigeminal neurons (Liu et al., 2024; Figures 1C, E). These events may consequently induce alterations in nervous tissue homeostasis (Tessarin et al., 2024). Furthermore, such microorganisms, their toxins, and probably EVs may travel through the lymphatic system, accumulate in the cerebral ventricles, and, together with the mechanisms discussed above, induce or potentiate neuroinflammation (Figures 1D, E).

When EVs derived from microorganisms access the CNS, they promote the activation of microglia and astrocytes, which subsequently produce cytokines, chemokines, and other inflammatory mediators (González-Sanmiguel et al., 2020). For instance, in PD, microglial cells become activated and exhibit increased release of IL-1β, IL-6, IL-8, IL-10, IL-12, IL-15, and TNF-α (Li et al., 2022; Tessarin et al., 2024). Activated microglia can further contribute to neurotoxicity by elevating levels of reactive oxygen species (ROS), which disrupt the function of multiple proteins and compromise cellular homeostasis (Li et al., 2022). The presence of BEVs contributes to the progression of Alzheimer's disease by promoting neuroinflammation and activating astrocytes via the complement C3/C3a receptor (C3/C3aR) signaling pathway, resulting in neuronal dysfunction, amyloid-β aggregation, and cognitive decline (Xie et al., 2023; Zhang et al., 2024). *In vitro* studies have demonstrated that *Helicobacter pylori* BEVs induce a reactive astrocyte phenotype through an NF-κB–dependent mechanism, thereby promoting neuronal injury (Palacios et al., 2023). Similarly, PI has been shown to promote astrocyte activation in the hippocampus and to increase the production of IL-1β, IL-6, and TNF-α, thereby contributing to neuroinflammation (Cafferata et al., 2024; Tessarin et al., 2024). In fact, no conclusive studies have reported that EVs derived from microorganisms associated with PI can reach the brain and induce or potentiate neuroinflammation and/or astrocyte and microglial activation. However, since microbiological analyses have demonstrated such interactions along the “gut–brain” and/or “mouth-brain axis”, it is possible to infer that a similar scenario may also occur in PI (Figures 1A–E).

### Eukaryotic extracellular vesicles and their potential role in central nervous system diseases

EVs comprise a heterogeneous population of naturally produced lipid bilayer particles released by both prokaryotic pathogens and eukaryotic cells (Effah et al., 2024). Eukaryotic EVs are commonly classified as exosomes (30–150 nm), microvesicles (100–1000 nm), and apoptotic bodies, which arise through endosomal pathways or by direct budding from the plasma membrane (Di Naro et al., 2025). These EVs are capable of exchanging components between cells, including nucleic acids, lipids, and proteins, thereby acting as signaling vehicles in normal cellular homeostatic processes (van Niel et al., 2018). However, these same vesicles may also play an important role in disease pathogenesis, including neurodegenerative conditions (El Andaloussi et al., 2013). This phenomenon has been demonstrated in Alzheimer's disease, in which amyloid-β peptides are released in association with exosomes, thereby contributing to pathogenic amyloid-β deposition in the brain (Bellingham et al., 2012). In addition, α-synuclein has been identified within EVs, suggesting a potential mechanism for the local propagation of Parkinson's disease pathology from enteric neurons to the brainstem and higher cortical centers (Emmanouilidou et al., 2010).

Banks et al. (2020) analyzed the capacity of EVs derived from murine macrophages, fibroblasts, and oral squamous cells, as well as human T cells, to cross the BBB. Using capillary depletion and intracerebroventricular injection methods, the authors reported that all EVs tested were able to cross the BBB, albeit with different influx rates (Banks et al., 2020). In addition, the possibility that EVs are internalized via endocytosis at one plasma membrane surface of endothelial cells, transported across the cell in membrane-bound vesicles, and released at the opposite membrane—a mechanism known as transcytosis—cannot be ruled out (Ramos-Zaldívar et al., 2022).

Thus, based on this brief overview, the possibility that EVs originating from endogenous cells present under PI conditions may reach the brain and alter homeostasis cannot be ruled out. Therefore, specific *in vitro* and *in vivo* experimental studies should be conducted to elucidate this hypothesis.

## Conclusion

In conclusion, microorganisms present in PD are also commonly found in PI, and several studies have demonstrated that vesicles released by periodontopathogens may be involved in the induction and/or potentiation of neuroinflammation. Thus, EVs from PI probably may disseminate systemically and reach the brain using different pathways, where they may contribute to neuroinflammatory processes. Similarly, eukaryotic EVs from PI sites may also alter CNS homeostasis. We emphasize that, to our knowledge, no experimental studies have been conducted to test the hypothesis that EVs originating from peri-implantitis sites may induce and/or potentiate neuroinflammatory conditions. Therefore, targeted studies are required to effectively elucidate this relevant issue.
